# Co-Chaperones in Targeting and Delivery of Misfolded Proteins to the 26S Proteasome

**DOI:** 10.3390/biom10081141

**Published:** 2020-08-04

**Authors:** Amanda B. Abildgaard, Sarah K. Gersing, Sven Larsen-Ledet, Sofie V. Nielsen, Amelie Stein, Kresten Lindorff-Larsen, Rasmus Hartmann-Petersen

**Affiliations:** 1 Department of Biology, The Linderstrøm-Lang Centre for Protein Science, University of Copenhagen, Ole Maaløes Vej 5, DK-2200 Copenhagen, Denmark; amanda.abildgaard@bio.ku.dk (A.B.A.); sarah.gersing@bio.ku.dk (S.K.G.); psd212@alumni.ku.dk (S.L.-L.); lindorff@bio.ku.dk (K.L.-L.); 2 Department of Biology, Section for Computational and RNA Biology, University of Copenhagen, Ole Maaløes Vej 5, DK-2200 Copenhagen, Denmark; svnielsen@bio.ku.dk (S.V.N.); amelie.stein@bio.ku.dk (A.S.)

**Keywords:** ubiquitin, proteasome, chaperone, co-chaperone, misfolding, protein quality control, protein stability

## Abstract

Protein homeostasis (proteostasis) is essential for the cell and is maintained by a highly conserved protein quality control (PQC) system, which triages newly synthesized, mislocalized and misfolded proteins. The ubiquitin-proteasome system (UPS), molecular chaperones, and co-chaperones are vital PQC elements that work together to facilitate degradation of misfolded and toxic protein species through the 26S proteasome. However, the underlying mechanisms are complex and remain partly unclear. Here, we provide an overview of the current knowledge on the co-chaperones that directly take part in targeting and delivery of PQC substrates for degradation. While J-domain proteins (JDPs) target substrates for the heat shock protein 70 (HSP70) chaperones, nucleotide-exchange factors (NEFs) deliver HSP70-bound substrates to the proteasome. So far, three NEFs have been established in proteasomal delivery: HSP110 and the ubiquitin-like (UBL) domain proteins BAG-1 and BAG-6, the latter acting as a chaperone itself and carrying its substrates directly to the proteasome. A better understanding of the individual delivery pathways will improve our ability to regulate the triage, and thus regulate the fate of aberrant proteins involved in cell stress and disease, examples of which are given throughout the review.

## 1. Introduction

Protein degradation is a fundamental part of the cellular machinery. An intricate set of protein networks known as the protein quality control (PQC) system coordinates the fate of non-native protein species. Since degradation of proteins is involved in a range of detrimental diseases [1,2,3], a need for a better comprehension of protein degradation has driven the field far. While our understanding of the degradation routes of misfolded proteins has improved, the exact mechanisms by which misfolded proteins are recognized and transferred to the proteasome remain elusive. Well-defined proteasomal shuttle factors are known to mediate interaction between the 26S proteasome and a large range of proteins involved in fundamental cellular processes [4,5]. However, in the case of the misfolded protein species that are handled by the PQC system, and likely constantly associate with molecular chaperones, our understanding of proteasomal delivery is more limited. In recent years, several studies have contributed important results and revealed an emerging role of co-chaperones. In this review, we summarize the current knowledge on how misfolded proteins are directed to the proteasome for degradation, and more specifically how molecular chaperones and their co-chaperones are involved in this process. Importantly, some misfolded proteins are refractory to degradation and the proteostasis system is known to decline during ageing, giving rise to accumulation of insoluble and aggregation-prone proteins that may be toxic and lead to disease. This has been reviewed in a number of excellent papers [6,7,8] and we will not discuss it further here.

## 2. Protein Misfolding

Proteins perform countless cellular functions that are essential to sustain proper cell function and organismal health. Therefore, the cell has evolved a complex molecular network of integrated processes to balance protein concentration, conformation, and subcellular localization in order to maintain protein homeostasis (proteostasis). The state of proteostasis is dynamically regulated by components of the proteostasis network, which facilitate fine-tuned control of the stability and functionality of the cellular proteome [9,10,11].

Most proteins must fold into a well-defined and specific three-dimensional structure, the native conformation, to achieve activity. Thus, protein function directly depends on the protein’s ability to reach this structure. Protein folding is a thermodynamically favorable process that is largely driven by intraprotein interactions, which must compensate for the tremendous conformational entropic penalty associated with the native conformation [12,13,14]. While many smaller proteins fold spontaneously, the great majority of newly synthesized proteins depend on the action of molecular chaperones to efficiently fold into their native conformation in the cellular environment [15]. Accordingly, chaperones are found in all domains of life and while the proteomes have expanded and become complex during evolution the molecular chaperones and co-chaperones have also expanded and become more abundant [16].

Most native conformations are only marginally stable with Δ*G_folding_* of about −5 to −10 kcal/mol under physiological conditions [17]. Consequently, proteins are extremely sensitive to translation errors, mutations, and chemical or physical stress conditions, which increase the risk of failure in reaching or preserving the native conformation. Such non-native conformations are susceptible to generating misfolded protein species [18].

Misfolded proteins often display reduced steady-state levels due to increased degradation rates, which in turn may result in loss-of-function phenotypes and protein misfolding diseases. Studies show that deletion of a single amino acid in the CFTR protein causes cystic fibrosis, due to misfolding and proteasomal degradation of the CFTR protein, even though the abnormal protein still retains some function [19,20,21]. We and others have found additional examples of protein misfolding diseases caused by an overzealous degradation system including Lynch syndrome, phenylketonuria and marble brain syndrome [1,2,3,22,23]. Intriguingly, it has been demonstrated that misfolded variants of the α1-antitrypsin protease inhibitor results in improper subcellular localization, which causes a loss-of-function phenotype. Consequently, the absence of the protease inhibitor in the correct subcellular compartment causes overactive proteases, which have severe effects [24,25,26]. Misfolded proteins can also have deleterious gain-of-function effects by forming toxic aggregates, which are often associated with neurodegenerative diseases such as Alzheimer’s and Parkinson’s disease [27]. Although the precise pathogenic mechanism remains unclear, the common hallmark of these neurodegenerative diseases is intra- and extracellular accumulation and deposition of insoluble aggregates in the brain, which correlate with progressive cellular dysfunction [28,29,30].

Thus, the extent and diversity of misfolded proteins and the numerous underlying mechanisms of protein misfolding diseases highlight the cellular demand for a highly coordinated, comprehensive and specific degradation system to fend against misfolded proteins. Accordingly, the cell has evolved an elaborate PQC system to monitor and maintain proteostasis [10,31].

## 3. Protein Quality Control

Two major elements of the cellular PQC are the ubiquitin-proteasome system (UPS) and the molecular chaperones and co-chaperones, which collaborate to decide the fate of aberrant protein species. In the following, we will briefly summarize these systems and highlight some examples showing that the UPS and the molecular chaperones are highly interconnected.

### 3.1. The Ubiquitin-Proteasome System (UPS)

The UPS manages the vast majority of cellular protein degradation [32]. The substrates of this system are diverse and include misfolded proteins [33], many short-lived proteins [34], but also long-lived proteins [32], as well as a large fraction of newly synthesized proteins that never reach their native conformation [35,36]. The broad yet specific degradation by the UPS is essential to avoid inadvertent degradation of cytosolic proteins, and relies on the regulatory protein ubiquitin and the elegant architecture of the 26S proteasome—a large multi-subunit protease complex.

Proteins are targeted to the proteasome through covalent linkage to the stable and conserved 76-residue protein ubiquitin [37,38,39,40], through lysine residues in target proteins [41]. Often, a chain of four or more ubiquitin molecules linked through internal lysine-48 residues is needed to direct a protein to the proteasome [42,43,44,45,46].

Ubiquitin conjugation requires three enzymes: an E1 ubiquitin-activating enzyme, an E2 ubiquitin-conjugating enzyme, and an E3 ubiquitin-protein ligase [44,47]. Following activation by the E1 and conjugation to the E2, the E3 transfers ubiquitin to a substrate protein, and thus provides the main substrate specificity of the ubiquitination cascade. Accordingly, the human genome is predicted to encode over 600 E3 ubiquitin-protein ligases [48]. A number of E3s have been connected with the PQC in both yeast and mammalian cells. For example, in yeast, Ubr1 and San1 have a well-characterized role in proteasomal degradation of misfolded proteins [49,50,51], while in mammals, carboxyl terminus of HSC70-interacting protein (CHIP) cooperates with the chaperone-system to ubiquitinate aberrant proteins [19,52,53,54].

Degradation of ubiquitinated proteins is mediated by the 26S proteasome, which is made up of a 20S core particle capped by one or two 19S regulatory particles. The regulatory particles further consist of two subcomplexes—the base and the lid. The lid partially covers the base, which connects to the core particle to gate the entry of substrate proteins [55,56,57]. Multiple intrinsic ubiquitin receptors on the regulatory particles mediate the recognition of ubiquitinated substrate proteins [58,59,60]. In addition, shuttle factors such as Rad23, Dsk2, and Ddi1 contain ubiquitin-like (UBL) and ubiquitin-associated (UBA) domains through which they mediate interaction between the proteasome [61,62,63,64,65] and ubiquitinated substrates [66,67,68] thus promoting substrate recognition and degradation [35].

Once bound by the proteasome, deubiquitinating enzymes, like the 19S subunit Rpn11 [69,70] or proteasome-associated Ubp6 [60,71] and Uch37 [59,72], remove the ubiquitin moiety to allow recycling of ubiquitin before degradation of the substrate. The complexity of the ubiquitin-chain determines the protein’s affinity for the proteasome and the rate of deubiquitination [73]. Following substrate recognition and removal of ubiquitin chains, the ATPase subunits of the base subcomplex promote protein unfolding and translocation into the cylinder-shaped core particle [74], which contains proteolytically active subunits. Access to the core-particle lumen is limited by α-subunits that form narrow openings at each end of the core particle [75,76]. This restricted access prevents inadvertent degradation of cytosolic proteins and allows for highly specific degradation. Through interaction with the α-subunits, the 19S particle ATPases mediate opening of the 20S gate, thereby allowing substrate entry to the proteolytic sites of the core particle [76,77]. Here, the proteolytically active subunits cleave the unfolded protein into short peptides [78,79], which diffuse to the cytosol for further cellular processing [32,80].

### 3.2. Chaperones as Substrate Recognition Factors in Protein Quality Control

Prior to ubiquitination and degradation, proteasomal substrates first need to be recognized. As misfolded proteins interact with molecular chaperones, these are often involved in the targeting of proteins to the UPS. Thus, the diverse classes of molecular chaperones are essential components in maintaining cellular proteostasis and interact with non-native proteins to promote, e.g., protein folding, compartmentalization, and degradation of their substrates. The focus in this review will be the heat shock protein 70 (HSP70) and HSP90 chaperone families due to their established roles in proteasomal degradation. However, other chaperones have also been linked to proteasomal degradation of misfolded proteins, including prefoldin [81] and the ubiquitin-specific ATPase complex known as Cdc48 in yeast or as p97 in humans [82].

#### 3.2.1. HSP70-Type Chaperones

The eukaryotic HSP70 chaperone family consists of multiple homologues that are either stress-induced (HSP70s) or constitutively expressed (HSC70s). These HSP70 homologues function in different cellular compartments, although the majority operate in the cytosol and nucleus, where the ubiquitin-proteasome system is localized. The HSP70 family members show functional diversity mediated by different substrate specificities, cooperation with co-chaperones and other chaperone systems, and subcellular localization [83,84,85,86]. However, it is important to point out that the binding cavity of HSP70 is conserved across family members and that differences between them are minor. In general HSP70-type chaperones have a similar structure and allosteric cycle, and thus show wide functional redundancy [87]. In contrast, a recent proteome-wide study by Ryu, et al. shows that there is essentially no substrate specificity overlap between the HSP70 and HSC70 families, except that they share newly synthesized proteins as common substrates [88].

**Figure 1:**
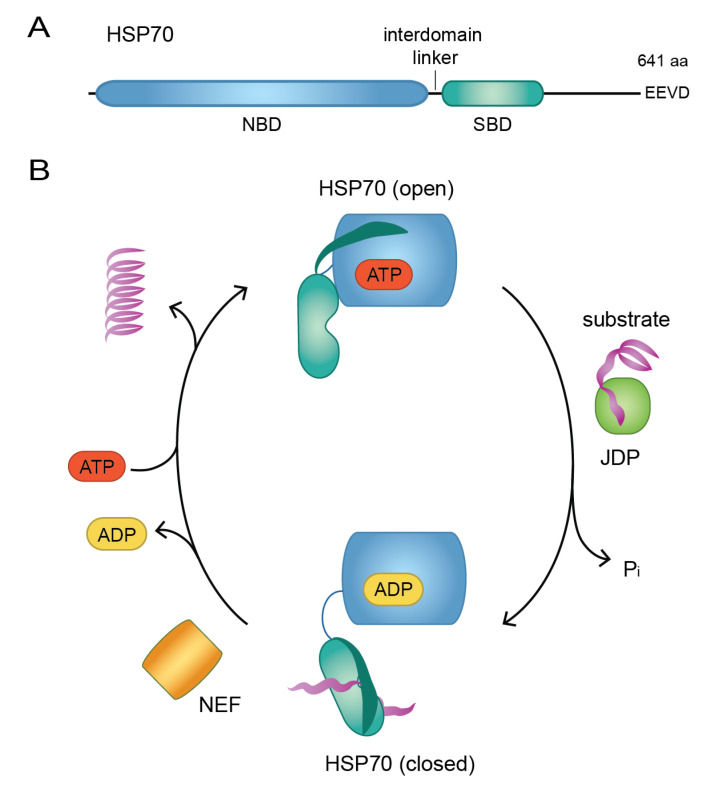
The substrate binding/release cycle of HSP70. (**A**) Schematic overview of the HSP70 domain structure. NBD: nucleotide-binding domain, SBD: substrate-binding domain, aa: amino acids. (**B**) The ATP-bound open conformation of HSP70 engages with a substrate-bound J-domain protein (JDP), which causes ATP hydrolysis and transfer of the substrate to HSP70 leading it to switch into its closed conformation. A nucleotide exchange factor (NEF) mediates ADP release, which permits ATP binding and results in opening of HSP70 and subsequent substrate release.

HSP70 consists of two major domains: an N-terminal nucleotide-binding domain (NBD), which is highly conserved between species, and a more variable C-terminal substrate-binding domain (SBD), which confers specificity (Figure 1A) [89,90]. The SBD comprises two subdomains; a compact β-sandwich that binds the substrate, which during the HSP70 cycle is encapsulated by an α-helical lid [91].

The foundation of HSP70 function is cycles of substrate binding and release (Figure 1B). These cycles are regulated by ATP binding and hydrolysis as well as substrate binding, and depend on the allosteric coupling of the NBD and SBD. When HSP70 is bound to ATP, the SBD is docked to the NBD [92], and the SBD is in an open conformation permitting substrate interaction. A J-domain protein (JDP), a family of HSP70 co-chaperones also known as HSP40s, promotes substrate binding to the SBD, resulting in disruption of the NBD-SBD interaction and stimulation of the NBD ATPase activity [92]. ATP hydrolysis induces a conformational change in the NBD, which is transmitted to the SBD and leads to stabilization of the HSP70-substrate complex [93,94]. At this stage, a member of the nucleotide exchange factor (NEF) family of co-chaperones can stimulate dissociation of ADP leading to a nucleotide-free state in which the NBD and SBD, like in the ADP-bound state, have limited interaction [92]. Binding of a new molecule of ATP to the NBD induces conformational changes resulting in extensive contacts between the SBD and NBD, opening of the SBD and subsequent substrate release [94,95,96]. To promote folding of substrates, this cycle of substrate binding and release is often repeated [97].

HSP70 is able to bind a wide variety of substrates with binding sites found on average once every 36 residues in a given protein [98]. HSP70 preferentially binds to extended protein conformations with sequences enriched in stretches of hydrophobic residues flanked by positively charged residues [98,99,100]. Hydrophobic residues are often buried in the core of folded proteins, and hence the specificity of HSP70 for these residues might promote recognition of unfolded or newly synthesized proteins, enabling HSP70 to shield intermolecular interactions that might result in aggregation [100]. A recent study proposed that HSP70 prefers basic over acidic residues because basic residues are more compatible with globular structure, but make the protein depend on HSP70 to suppress aggregation [101]. In addition, others have suggested that the binding mechanism of HSP70 is flexible, allowing it to accommodate partially folded regions of proteins [102]. HSP70-bound substrates have been shown to be largely unfolded and have an expanded conformation, potentially due to multiple HSP70s binding the same substrate [103,104]. Meanwhile, others find that substrates can retain some secondary structure outside the HSP70-binding region [105], or have partially-folded and near-native structures [106].

HSP70 is proposed to bias the substrate’s folding pathway by disrupting long-range interactions, while allowing secondary structure to form [107] as the substrate samples the conformational space while bound to HSP70 [105,108]. In this way, HSP70 enables local structures to form prior to the establishment of long-range interactions that can form following release from HSP70 [107]. However, association with HSP70 does not always lead to protein folding, and successive cycles on HSP70 can instead lead to transfer of the substrate to downstream chaperone or degradation systems. The exact mechanism for HSP70- and HSP90-promoted protein folding was addressed by Morán Luengo et al., saying that the hydrophobic binding by HSP70 blocks substrate folding, whereas subsequent binding by HSP90 releases this blockage allowing the substrate to refold [109]. Consequently, inhibition of HSP90 and thus substrate refolding leads to HSP70-linked proteasomal degradation of the substrate.

#### 3.2.2. HSP90-Type Chaperones

HSP90 is a highly conserved molecular chaperone that cooperates with HSP70 in protein folding [110,111,112]. HSP90 comprises three domains: an N-terminal domain, a middle domain, and a C-terminal domain. Together these three domains enable HSP90 to bind and hydrolyze ATP, associate with clients, and dimerize [113]. The HSP90 homodimer cycles between an open V-shaped conformation and a closed state regulated by ATP [114]. In addition, the cycle is regulated by HSP90 co-chaperones, that also to some degree mediate client specificity [115]. HSP90 clients are predominantly metastable proteins such as kinases, transcription factors, and E3 ubiquitin ligases. In addition, the distinction between HSP90 clients and nonclients might partly be based on the stability of the client protein, at least for protein kinases [116].

Compared to HSP70, HSP90 preferentially binds late folding intermediates containing a higher degree of structure [117]. This client preference is facilitated by the large substrate-binding interface of HSP90 [118]. Accordingly, HSP90 often operates downstream of HSP70 as a regulator of client activity by facilitating the final steps of protein folding, assembly of multiprotein complexes, and binding of ligands to substrates [114]. Moreover, like other molecular chaperones, HSP90 acts as a buffer upon environmental stress conditions by supporting folding and activation of client proteins. Accordingly, a recent study found that HSP90 has evolved to support growth in multiple stress conditions, and thus to provide cellular robustness [119].

#### 3.2.3. Chaperones in the Heat Shock Response

As previously mentioned, environmental or pathophysiological conditions can increase the cellular abundance of non-native proteins and thus the demand for components of the PQC network. Whilst the cells constitutively express various chaperones and co-chaperones to manage proteostasis under normal conditions, many heat shock proteins are expressed further or exclusively under conditions of proteotoxic stress such as during a heat shock [120]. At the heart of this response, is the transcription factor heat shock factor 1 (HSF1). HSF1 is regulated by chaperone binding, nucleo-cytoplasmic shuttling, numerous post-translational modifications and by the transition from monomer to a transcriptionally active homotrimer [121]. In yeast, Hsf1 activation also appears to involve cytosolic acidification [122]. Importantly, chaperone complexes of both HSP90 and HSP70-HSP40 associate with HSF1 [123,124,125] and upon proteotoxic stress, the need for chaperones to bind misfolded and newly translated proteins will titrate them away from HSF1 allowing HSF1 to initiate transcription of *HSP* genes [126,127]. The upregulation of *HSP* genes ensures a negative feedback loop, and once the proteotoxic load is under control, HSPs will again be free to bind HSF1 and inactivate it [128]. Simultaneously, HSF1 acts as a repressor to reduce protein synthesis during heat shock [121,129,130], explaining why activation of the HSF1 response results in growth arrest in several species [131].

#### 3.2.4. Molecular Chaperones and Protein Degradation

The intrinsic ability of HSP70 and HSP90 to associate with non-native proteins makes them ideal to serve as recognition factors for the UPS. Accordingly, both HSP70 and HSP90 mediate proteasomal degradation of substrate/client proteins [132,133,134,135,136]. Indeed, the involvement of chaperones in protein degradation is widespread [137]—e.g., chaperones mediate the degradation of the disease-causing CFTR variants previously mentioned [138,139].

To facilitate degradation, molecular chaperones cooperate with E3 ubiquitin-protein ligases to promote substrate ubiquitination [134,140,141,142,143]. Moreover, HSP70 and HSP90 interact with the 26S proteasome and deliver ubiquitinated proteins destined for degradation [144,145], making them key players in protein triage decisions and in maintaining the delicate balance between protein folding and degradation [146,147]. Importantly, like all cellular roles of molecular chaperones their role in degradation is heavily regulated by co-chaperones, as described in the following sections.

## 4. Co-Chaperones Decide the Fate of HSP70-Bound Substrates

### 4.1. Regulation of Chaperone Activity

In order to carry out the crucial role of detecting and directing substrates to the proteasome, the HSP70 chaperone family depends on assistance from a vast network of co-chaperones. HSP70 function relies on the action of regulatory JDPs or HSP40s, which both shuttle substrates to HSP70 and catalyze the slow intrinsic ATP hydrolysis rate of HSP70, thereby facilitating trapping of bound substrates.

The JDP family is defined by the presence of a J-domain: a 70 residue alpha-helical hairpin structure with a flexible loop, carrying a conserved and functionally crucial histidine-proline-aspartic acid motif [148]. Through the J-domain, DnaJ binds at the interfaces of the NBD, the SBD and the interdomain linker of *E. coli* HSP70 DnaK, thus inducing ATP hydrolysis and transfer of the substrate to DnaK [87,149,150].

Humans encode 34 classical JDPs and 7 with modified J-domains [151]. This diverse group of proteins is traditionally divided into three classes (I, II and III) depending on their resemblance to the canonical *E. coli* JDP, DnaJ [85]. DnaJ contains an N-terminal J-domain, a G/F-rich domain, a cysteine-rich zinc finger domain and three C-terminal binding domains (CBDs) [152]. Through hydrophobic pockets formed by the CBDs DnaJ binds hydrophobic and aromatic residues in the substrate. This is an important step in substrate maturation, since binding of DnaJ partly unfolds the substrate and thus increases the number of accessible HSP70 binding sites on the substrate [153]. Moreover, JDP binding prevents substrates from aggregating. For example, the human JDP ERdj3 is essential in Ig assembly, as it prevents aggregation of the light chain molecules, by binding unfolded Ig light chains before they are handed over to the ER-localized HSP70 BiP for folding assistance [154].

Specific structural features determine the JDPs’ substrate preferences and functions. For example, while the G/F-rich region is essential for substrate binding by DnaJ [155], a serine-rich region of human DNAJB6 and DNAJB8 is necessary for binding of polyglutamine substrates [156]. Recently, a study showed that the yeast Hsp40 Apj1 mediates disaggregation and turnover of proteins aggregated within the nucleus [157]. Moreover, JDPs direct substrates to distinct E3 ligases for degradation, as in yeast, where Ydj1 is involved in Ubr1/San1-linked degradation, and Sis1 is involved in degradation through Doa10/Hrd1 [158]. In both yeast and mammalian cells, the main E3 responsible for the increased ubiquitination of cytosolic proteins after heat shock appears to be Rsp5/NEDD4-L [159]. Interestingly, this ubiquitination was also found to be dependent on Ydj1, which interacts with Rsp5 upon heat shock. A two-step substrate recognition mechanism has been proposed, requiring Ydj1 for initial recognition of the misfolded protein after which natively buried PY motifs bind to the WW repeats of Rsp5 and allow it to ubiquitinate the substrate [159]. The subsequent degradation of the ubiquitinated, misfolded proteins is dependent on the deubiquitinase Ubp2, presumably because it removes ubiquitin chains allowing threading of the substrate into the proteasome [160].

Thus, the presence of specific JDPs determines the fate of specific target substrates. Accordingly, a yeast-based study recently showed how the HSP70 homologue Ssa2 has significantly larger affinity for Ydj1 than its paralogue Ssa4, which may be the reason for the pronounced ability of Ssa2 to assist maturation of Hsp90 substrates [161]. Since JDPs regulate which substrates are presented to HSP70, they are essential components of the chaperone network. For example, DNAJB6 and DNAJA1 were recently shown to have opposing effects on the levels of cellular polyglutamine aggregates, meaning their cellular abundances alone modulate aggregate formation [162]. Moreover, the levels of co-chaperones DNAJA1 and DNAJA2 regulate CHIP/HSP70-mediated degradation of mutant CFTR: while both co-chaperones are needed for CFTR folding, increased levels of DNAJA2 promote degradation of CFTR [163]. Other studies show how inhibition of JDPs can regulate chaperone activities directly—a mechanism that can be used to treat chaperone-related diseases. For example, in the attempt to treat HSP90-related diseases such as prostate cancer, Alzheimer’s disease and cystic fibrosis, inhibition of the HSP90 JDP, Aha1, was found to specifically inhibit a subset of disease-linked HSP90-activities [21,164]. Indeed, this observation fits with an extensive study of HSP90 co-chaperones that find that the fate of specific clients depends on the presence of different co-chaperones [165]. Another study on chaperone regulation found that the underlying mechanism of the inherited muscular dystrophy disease LGMD1D is due to increased affinity between HSP70 and a mutated variant of the JDP DNAJB6, thereby locking HSP70 to this complex, preventing it from completing its cellular tasks. By inhibiting the interaction between HSP70 and DNAJB6, HSP70 molecules were released, relieving disease models from symptoms of muscular dystrophy [166].

Once the substrate is bound to HSP70, the chaperone requires assistance from NEFs to facilitate the release of ADP, thus allowing ATP-binding and subsequent substrate-release. Since NEFs have the ability to release misfolded proteins from the protective grip of chaperones, they play a critical role in deciding the fate of the substrate.

There are four types of NEFs with completely distinct structures and modes of action [87]. In prokaryotes and mitochondria, the GrpE-type carry out the NEF activity, while eukaryotes express Armadillo, HSP110 and BAG-type NEFs. Humans encode two mitochondrial GrpE, two Armadillo, four HSP110 and six BAG-type NEFs [167].

In *E. coli*, GrpE dimerizes through an N-terminal domain and interacts with DnaK through a β-sheet domain, which it inserts into the DnaK NBD. This forces the opening of the NBD and stimulates ADP release. Moreover, GrpE performs substrate mimicking and inserts a disordered N-terminal domain into the SBD of DnaK. It thereby competes with the HSP70-bound substrate and further promotes substrate release [168,169,170]. The yeast Armadillo-type NEF, Fes1, and its human orthologue HSPBP1 also use a disordered N-terminus to perform substrate mimicking [171]. Armadillo-type NEFs stimulate ADP release with their C-terminal domain of four Armadillo repeats, which wraps around the HSP70 NBD and forces it open [167,172]. Deletion of Fes1 has been found to inhibit degradation of some HSP70-bound substrates [173], showing that NEFs have distinct cellular functions, and that their individual cellular level can determine the fate of a given substrate.

The HSP110-type NEFs are a subfamily of heat shock proteins within the HSP70 superfamily, and thus share structural similarities with HSP70 [174,175,176]. Once activated by ATP [177], the HSP110 NBD wraps around the HSP70 NBD, which imposes the ADP-releasing conformational change [87,178,179]. The NEF activity is the key cellular role of HSP110 [180]. However, as HSP110 is able to bind unfolded proteins through its SBD [181], its presence prevents protein misfolding and aggregation. Accordingly, HSP110 expression is induced upon stress signaling [120] and currently, an inhibitor of HSP110, foldamer 33, is being tested as a potential anticancer drug that prevents HSP110-mediated stabilization of the oncogenic protein STAT3 [175]. A recent study shows that HSPH2, a human HSP110 homologue, is essential for the cellular role of the HSP70 HSPA1A [182]. The HSP70 homologues HSPA1L and HSPA1A have opposing effects on the aggregation-prone substrate, (SOD1)-A4V, and promote aggregation or dissolution, respectively. The differential affinities for two co-chaperones, HSPH2 or Hop, seem to confer this difference, with HSPH2 mediating dissolution of aggregates through HSPA1A [182].

**Figure 2:**
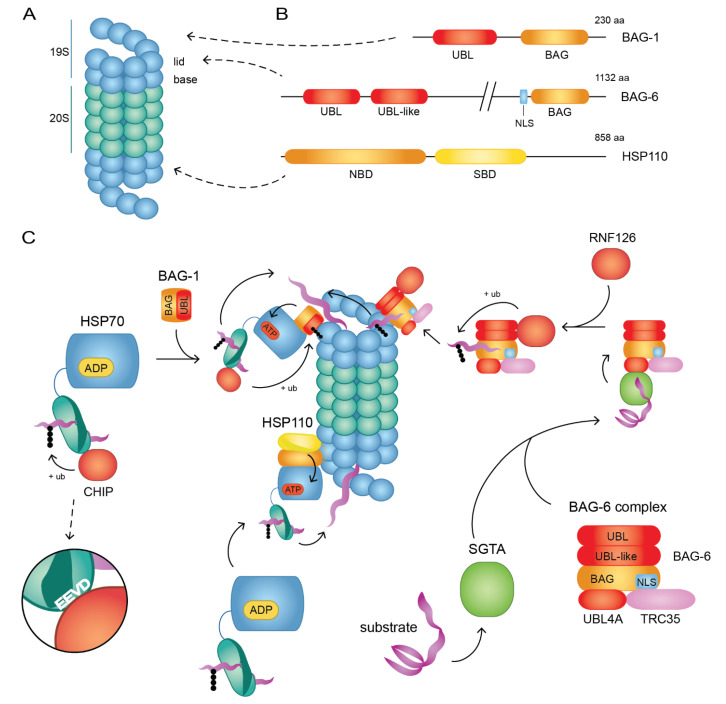
Delivery pathways of misfolded proteins to the 26S proteasome. (**A**) The 26S proteasome with one catalytic 20S particle (green) and two regulatory 19S particles (blue) each containing a lid and a base subcomplex. (**B**) Schematic overviews of BAG-1, BAG-6 and HSP110 domain structures, which bind the regulatory 19S particles (arrows). UBL: ubiquitin-like, BAG: Bcl2-associated athanogene, NLS: nuclear localization signal, NBD: nucleotide-binding domain, SBD, substrate-binding domain, aa: amino acids. (**C**) Delivery pathways to the 26S proteasome. Left: CHIP binds HSP70 through its C-terminal EEVD motif and ubiquitinates the bound substrate. The substrate-HSP70-CHIP complex engages with BAG-1, which connects the complex to the proteasome through binding of the 19S particle through its UBL domain. The binding is strengthened by CHIP-mediated ubiquitination of the UBL-domain. Substrate release at the proteasome is mediated by BAG-1. Middle: HSP110 binds the 19S regulatory particle, where it associates with a substrate-HSP70 complex. NEF activity by HSP110 releases ADP from HSP70, which in turn mediates substrate release. Right: The substrate-bound JDP, SGTA, binds the BAG-6 complex consisting of BAG-6, UBL4A and TRC35. This promotes transfer of the substrate to BAG-6. Next, RNF126 binds the UBL-domain of BAG-6 and ubiquitinates the substrate. The BAG-6 complex binds the 19S particle of the proteasome, thus enabling substrate degradation.

Lastly, the BAG-type NEFs are defined by a HSP70-binding Bcl2-associated athanogene (BAG) domain in their structure [183]. A structural analysis of the BAG-HSC70 complex revealed that the BAG domain forms a three-helix bundle and binds the HSP70 NBD, which induces a conformational change similar to that induced by GrpE [184]. The six human BAG proteins contain different accessory domains, which specify their cellular roles [185]. Of interest to this review, BAG-1 and BAG-6 have been linked to UPS-mediated degradation of misfolded proteins. Importantly, they both contain N-terminal UBL-domains that bind the 19S regulatory particle of the 26S proteasome (Figure 2A,B), while BAG-6 also contains a UBL-like domain related to substrate binding [185,186].

Alterations in the levels of certain co-chaperones can give rise to a heat shock/HSF1-mediated stress response. For example, deletion of Fes1 induces a strong, constitutive heat shock response in yeast [173]. Thus, the induction of multiple chaperones appears to compensate for the PQC defects associated with the deletion of Fes1. Curiously, the deletion of HSP110-type NEFs Sse1 and Sse2 does not give rise to a heat shock response. However, cells lacking Sse1 accumulate ubiquitin conjugates both at 25 °C and under heat shock [173]. Deletions of the BAG-domain containing NEFs Bag101 and Bag102 in *Schizosaccharomyces pombe* display no obvious growth phenotypes [187]. However, overexpression of Bag101, and perhaps other NEF-type co-chaperones, gives rise to a HSF1-mediated stress response that results in a growth defect, and a transcriptional response similar to that observed upon deletion of the HSP70 Ssa2 or the JDP Mas5 (orthologue of yeast Ydj1) [188]. Since, *S. pombe* Ssa2 and Mas5 are responsible for binding and inactivation of HSF1 under unstressed conditions [189], Bag101 therefore likely releases HSF1 from the Ssa1-Mas5 chaperone and initiates the observed transcriptional response [188].

### 4.2. Nucleotide Exchange Factors Direct Substrates to the Proteasome

#### 4.2.1. Degradation through BAG-1

Delivery of some misfolded proteins to the proteasome occurs through BAG-1 in collaboration with HSP70 and CHIP (Figure 2C). This pathway is well-established in human cells, and mediates degradation of toxic and aggregation-prone mutant huntingtin [190], immature BCR-ABL oncoproteins [191] and misfolded hERG potassium channels [192].

Notably, CHIP acts both as a co-chaperone and an E3 ubiquitin ligase [53]. First, CHIP binds the C-terminal EEVD motif of HSP70 through a tetratricopeptide repeat (TPR) domain [193]. The high conformational flexibility of the substrate-HSP70-CHIP complex permits substrate presentation to CHIP [194]. Members of the UBCH5 E2 family carry ubiquitin molecules to CHIP, which ubiquitinates the substrate [54]. In an unrelated binding event, BAG-1 binds the ternary complex at the HSP70 NBD through its C-terminal BAG domain [184,195]. Then, BAG-1 acts as a shuttle factor and transports the BAG-1-substrate-HSP70-CHIP complex to the proteasome, by binding the 19S regulatory particle through its N-terminal UBL domain [53,195]. Specifically where on the proteasome BAG-1 binds is currently unknown, however, it likely binds subunits also bound by other UBL-domain shuttle factors, such as the Rpn1 (Elsasser et al., 2002) and Rpn2 subunits [196]. Accordingly, in *S. pombe* the BAG-1 orthologue Bag101 [187] competes with the Rad23 orthologue Rhp23 for proteasome binding [188].

Following proteasomal binding, CHIP strengthens the BAG-1/proteasome interaction by attaching a lysine-11 ubiquitin chain to BAG-1 [197]. Simultaneously, BAG-1-binding leads to nucleotide exchange within HSP70 and thus promotes substrate release [184,198,199]. Through this intricate mechanism, the unfolded and ubiquitinated substrate is brought in close contact with the proteasome leading to its degradation.

Regulation of the cellular ratios of chaperones and co-chaperones is important in order to avoid substrates becoming trapped for an unfavorably long time, which would prevent refolding and occupying the chaperone, or that they dissociate too quickly, which would increase the risk of substrate aggregation [85,198,200]. In line with this, the BAG-1/HSP70/CHIP degradation pathway strongly depends on the available concentrations of BAG-1 and CHIP. Accordingly, CHIP acts as a switch on HSP70 since increased cellular concentration of CHIP amplifies proteasomal degradation of HSP70-bound substrates [52]. Another important regulator of BAG-1 is the co-chaperone HSC70 interacting protein (Hip), which has antagonistic functions to BAG-1 [199,201,202], and indeed all types of NEFs [203]. Competitively to BAG-1, Hip binds the ADP-bound NBD of HSP70 through a TPR repeat, which stabilizes the ADP-bound state and thereby inhibits substrate release, directly opposing the actions of NEFs. In addition, the human Armadillo-type NEF HSBP1 only inhibits the E3 activity of CHIP, by binding the CHIP-HSP70 complex. By interfering with the E3 activity of CHIP, the degradation of misfolded CFTR protein species is inhibited [204,205].

An alternate CHIP-linked degradation pathway involves the E3 ubiquitin ligase parkin, which is a UBL domain protein involved in regulated protein degradation of the membrane protein PaeI receptor (PaeI-R). Unfolded PaeI-R binds to parkin, which further associates with HSP70 and CHIP [205,206]. In this complex, parkin acts as a shuttle factor and binds directly to the Rpn13 subunit of the 19S particle of the proteasome, thus mediating degradation of PaeI-R [207].

Notably, CHIP also binds HSP90 and thus targets HSP90-bound substrates for proteasomal degradation in the same structurally flexible fashion seen for the substrate-HSP70-CHIP complex [194]. For example, the CHIP/HSP90 degradation pathway targets the hyper-phosphorylated tau protein, which is known to cause Alzheimer’s Disease [132], however, no proteasomal delivery factor is known for this pathway [194].

The degradation of several chaperone clients is not affected in CHIP-deficient mammalian cells [208], indicating functional overlap between CHIP and other E3s. Accordingly, overlapping substrate specificity between different E3s has also been observed in yeast cells [158,187,209], and suggests that BAG-1 may collaborate with other E3s [187]. For example, BAG-1 plays a role in inherited cardiac arrhythmia as it targets misfolded variants of the hERG potassium channel for ER-associated degradation (ERAD) in an indirect and UBL-domain independent manner [192]. While HSP70 assists folding of cytosolic domains of the hERG potassium channel, interaction with BAG-1 releases hERG from HSP70, and thus prevents refolding. In this case, release from HSP70 was found to promote substrate binding to the ER-associated E3 TRC8, leading to its degradation through ERAD. Thus, BAG-1 in this case indirectly switches the fate of misfolded hERG from HSP70-dependent folding to degradation.

#### 4.2.2. Degradation through BAG-6

The human BAG-6 protein, also known as Scythe or BAT3, plays an essential role in the quality control of tail-anchored (TA) membrane proteins [210], cytosolic mislocalized proteins [211], misfolded ERAD substrates [212], and newly synthesized defective proteins [213]. For example, BAG-6 contributes to the degradation of the uncleaved human leukocyte antigen (HLA-A) protein [214] as well as the aggregation prone prion protein (PrP) in the cytosol [215]. Consistent with the role of BAG-6 in the degradation of mislocalized membrane proteins from the ER under native conditions, BAG-6 is also crucial for the increased degradation that is induced upon ER stress [216].

BAG-6 does not act as a NEF in this pathway but as a chaperone itself. Like other chaperones, it plays an important role in triaging its substrates. While its folding capability is minor, BAG-6 can act as a holdase and prevent aggregation of TA transmembrane proteins [210], and keep ERAD-substrates soluble before further processing by p97 [212,217].

Interestingly, analyses of the BAG-6 protein reveal that the structure of its BAG domain differs substantially from other BAG proteins [218,219], causing a weak in vivo interaction with HSP70 [220]. Thus, BAG-6 probably largely operates in an HSP70-independent manner as part of a heterotrimeric complex consisting of transmembrane domain recognition complex of 35 kDa (TRC35) and ubiquitin-like protein 4A (UBL4A) [210] (Figure 2C). BAG-6 plays the key role in the BAG-6-TRC35-UBL4A complex (BAG-6 complex below) and binds substrates through its N-terminus [213]. Meanwhile TRC35 retains the complex to the cytosol, by covering the nuclear localization signal within the BAG-6 sequence [212,221], as well as mediating interaction with the targeting factor TRC40, which facilitates insertion of the substrate to the ER-membrane [222,223]. UBL4A binds the unconventional BAG domain of BAG-6 with high affinity [218] and forms the link between the BAG-6 complex and the co-chaperone small glutamine-rich tetratricopeptide repeat containing protein alpha (SGTA), which binds to the UBL domain of UBL4A through its N-terminus [224].

In this pathway (Figure 2C), SGTA detects newly synthesized TA proteins or similar substrates with long hydrophobic stretches in the cytosol [225]. BAG-6 has a similar substrate preference [210,215] and the BAG-6 complex transfers the SGTA-bound substrates to TRC40 for membrane insertion [222]. If the substrate is for some reason unfit for membrane insertion, e.g., due to misfolding, the substrate remains in the SGTA/BAG-6 cycle. This leads to ubiquitination of the substrate by the E3 ubiquitin ligase RNF126, which binds the UBL-domain of BAG-6 through its N-terminus [211]. Moreover, reubiquitination by the BAG6/RNF126 pathway was recently found to be an essential step in targeting deubiquitinated membrane-proteins for proteasomal degradation [226]. Next, the BAG-6 complex binds to the Rpn10 subunit of the proteasome’s regulatory 19S particle, thus promoting degradation of the substrate [213,219,227]. Moreover, UBL4A was recently shown to facilitate peptide entry to the proteasome [228], which is likely to enhance the degradation efficiency of substrates bound by the BAG-6 complex.

The co-chaperone SGTA has been shown to bind Ydj1 in yeast [229] and also holds a TPR repeat, with which it can bind HSP70 and the Rpn13 subunit of the 19S regulatory proteasome particle [230,231,232], suggesting a more extensive role for SGTA in PQC. Indeed, binding of SGTA to Rpn13 has been found to increase cellular levels of substrate proteins [230], indicating that SGTA could oppose the role of BAG-6 in substrate degradation.

#### 4.2.3. Degradation through HSP110

In a recent study, the yeast HSP110 Sse1 and its paralogue Sse2 were found to be involved in delivery of proteasome substrates in collaboration with yeast HSP70 orthologues Ssa1-4 [233]. The results show that HSP110 interacts with the regulatory 19S particle of the proteasome and that inhibition of HSP110 causes accumulation of HSP70-bound proteasome substrates, which include both ubiquitin-modified and unmodified substrates. The authors suggest a mechanism where HSP110 first binds the 19S particle and then recruits the substrate-HSP70 complex (Figure 2C). At the proteasome, HSP110 promotes HSP70 nucleotide exchange, which in turn mediates substrate release and degradation. The study further supports a general role of HSP70-binding as being one of the first steps towards proteasomal degradation of a misfolded protein, and that subsequent binding by HSP110 is the defining step leading to degradation. However, this role for HSP110 has so far only been established in yeast, and the extent to which the four human HSP110 proteins are involved in degradation is currently unknown.

Importantly, these observations fit well with previous studies showing that HSP110 contributes to the triage of HSP70-bound substrates. To this end, ubiquitination of HSP70-bound substrates has been found to depend on Sse1, and upon inhibition of HSP90, Sse1 seems to stimulate degradation [234].

### 4.3. Future Questions for Understanding the Role of Co-Chaperones as Shuttle-Factors for Misfolded Proteins

As reviewed in this paper, chaperones and co-chaperones are broadly involved in the degradation of misfolded proteins. So far, BAG-1, BAG-6 and HSP110 have been identified as proteasome-interacting co-chaperones and direct mediators of proteasomal degradation of misfolded proteins. However, a line of questions remains unanswered. For instance, how do these factors differ in their substrate selection? Do the co-chaperones contribute to the substrate specificity and are the E3s also directly involved? Moreover, at which stage are the target proteins ubiquitinated? In case of the yeast E3 San1 it is clear that this particular PQC E3 directly targets misfolded proteins [50,235], and although a number of mammalian E3s display San1-like sequence properties [236] it is currently unclear to what extent other PQC E3s are involved in target selection. However, even in the case of San1, it has been shown that certain San1 substrates engage with chaperones prior to San1-catalyzed ubiquitination [142,237], and perhaps after ubiquitination the targets may still be shuttled to the proteasome via chaperones and released in a BAG and/or HSP110-dependent manner [187]. In addition, in most cases the structural and/or sequence properties of the regions that are recognized by the PQC E3s are not well defined. Although significant screening efforts have identified many of these so-called PQC degrons [238,239], and a few have been characterized in more detail [238,239,240,241], no clear pattern has yet emerged.

In both BAG-1 and HSP110-linked degradation, substrate selection appears to depend largely on the HSP70-substrate interaction, suggesting that BAG-1 and HSP110 functionally overlap and trigger degradation of similar substrates. However, whether the NEFs themselves further regulate substrate specificity is not yet clear. In addition, the NEFs may hold different nucleotide exchange efficiencies and proteasome affinities or be subject to regulation by post-translational modification or other mechanisms. Interestingly, a study recently found that binding of UBL-domain proteins to the proteasome activates the proteasome and stimulates its proteolytic activity [228]. Potentially, this may affect the efficiency by which the BAG domain proteins stimulate degradation compared to HSP110.

As for BAG-6, the substrate specificity depends on the binding preference of BAG-6 itself, which favors longer hydrophobic stretches over the shorter regions that are typically recognized by HSP70 [211]. Hence, the extent of substrate misfolding may ultimately decide which degradation route is followed. In this regard, it is also noteworthy that the lid covering the substrate-binding region in HSP70 is only slightly closed when bound to partially folded molten globule-like proteins and fully closed when binding to a short hydrophobic peptide, and conceivably such differences might also decide the fate of the bound substrate [87,242].

Finally, in addition to the co-chaperones, a number of studies have also connected the established proteasomal substrate shuttles Rad23 and Dsk2 with the degradation of misfolded proteins [243]. Clearly, a ubiquitinated misfolded protein will likely require these shuttle proteins for efficient proteasomal degradation. However, early studies revealed that Rad23 and Dsk2 contain Sti1-like repeat sequences similar to that found in Hip [244], suggesting a more direct link with PQC. More recently, it was shown that upon heat shock, ubiquitinated substrates increase and coprecipitate with HSP70 and Dsk2 [233], and that Dsk2 is important for shuttling nuclear misfolded and ubiquitinated substrates to the proteasome [158].

Thus, clearly the many unanswered questions above warrant more research into how the PQC machinery regulates the degradation of misfolded proteins, and the emerging role of co-chaperones as key players in proteasomal delivery will likely develop in the coming years. As mentioned throughout this review, the actions by the PQC are involved in many widespread and detrimental diseases caused by genetic and environmental factors. Importantly, a deeper understanding of how the PQC triages the fate of misfolded proteins and regulates their delivery at the 26S proteasome would surely increase our chances of diminishing the consequences of these diseases.
